# Improving Thermo-Oxidative Stability of Nitrile Rubber Composites by Functional Graphene Oxide

**DOI:** 10.3390/ma11060921

**Published:** 2018-05-30

**Authors:** Rui Zhong, Zhao Zhang, Hongguo Zhao, Xianru He, Xin Wang, Rui Zhang

**Affiliations:** 1 State Key Laboratory of Oil and Gas Reservoir Geology and Exploitation & School of Materials Science and Engineering, Southwest Petroleum University, Chengdu 610500, China; 201621000035@stu.swpu.edu.cn (R.Z.); zzismrz@163.com (Z.Z.); 2 Petrochemical Research Institute, PetroChina, Lanzhou 730060, China; tongyongyj@126.com; 3 Institut für Physik, Universität Rostock, Albert-Einstein-Str. 23-24, 18051 Rostock, Germany; rui.zhang@uni-rosotck.de

**Keywords:** graphene oxide (GO), *p*-phenylenediamine (PPD), nitrile rubber (NBR), thermo-oxidative stability

## Abstract

Graphene oxide (GO), modified with anti-aging agent *p*-phenylenediamine (PPD), was added into nitrile rubber (NBR) in order to improve the thermo-oxidative stability of NBR. The modification of GO and the transformation of functional groups were characterized by Fourier transform infrared spectroscopy (FTIR), Raman, and X-ray diffraction (XRD). Mechanical performances of NBR composites before and after the thermo-oxidative aging were recorded. The results of dynamic mechanical analysis (DMA) show an increased storage modulus (G’) and a decreased value of area of tan δ peak after introducing modified GO into NBR. It indicates that filler particles show positive interaction with molecular chains. The thermo-oxidative stability of composites was investigated by thermogravimetric analysis (TG) and differential scanning calorimetry (DSC). Then, the thermo-oxidative aging kinetic parameters were obtained by the Flynn–Wall–Ozawa (FWO) equation. The results of aging tests show that the thermo-oxidative stability of rubber matrix increases obviously after introducing GO–PPD. In addition, mechanical properties (tensile strength and elongation at break) of both before and after aged NBR/GO–PPD composites were superior to that of NBR. This work provides meaningful guidance for achieving multifunction thermo-oxidative aging resistance rubber composites.

## 1. Introduction

The ability of an elastomeric composition to apply at elevated temperature for a long time is a paramount requirement in many fields, such as tire [1], sealing [2], viscoelastic damping materials [3], etc. Due to its superior oil resistivity, nitrile rubber (NBR) has been widely applied for production of oil seal devices. Generally, in real conditions, NBR elastomer components are exposed to various fluids, such as crude oil, various petroleum products, gas condensates, natural gas, CO_2_ and H_2_S. Furthermore, NBR elastomer products usually work at an elevated temperature (up to 120 °C) and high pressure, at the same time [4,5,6]. However, the existence of unsaturated isolated double bonds makes it easy for NBR to be decomposed by oxygen, especially under conditions of high heat and pressure [4]. For this case, many nanofillers have been applied to improve thermal stability of NBR, including silica [7], carbon nanotube [8], Fe_3_O_4_ [9], graphene, etc. [10].

However, nanofillers also change the mechanical properties of NBR during enhancing thermal stability. Graphene, a single-atom-thick sheet of carbon atoms densely packed in a honeycomb crystal lattice, has been the subject of considerable interest and study, because of its intriguing and outstanding physical properties [10]. As expected, graphene holds promising applications in rubber composites, since it could endow rubber with reinforcement and functional properties at very low loading [11]. Moreover, it is believed that the superior properties of the graphene nanocomposites are not only because of the nanoscale dispersion of graphene within the rubber matrix, but are also the result of a strong interaction between rubber molecular chains and graphene [11,12,13]. Graphene oxide (GO), because of its laminated structure with the function of oxygen barrier, is popularly used in flame-retardant materials [14,15]. In addition, GO has been reported that it could improve the thermostability of polymers, including epoxy resin [16], natural rubber [17], cellulose [18], etc. Meanwhile, graphene oxide could construct complex networks in polymers, which leads to an increase in physical crosslink points. In conclusion, the mechanical properties and thermostability could be improved by incorporating GO in NBR at the same time [15,16,17]. Unlike the ideal graphene, GO was easily further modified for wider utilization [18,19,20].

It has been reported that PPD could effectively improve the performance of supercapacitors [21,22]. Due to the existence of antiaging amino group, PPD has been widely applied to improve the thermostability and anti-aging property of polymers, including natural rubber [23], polyisoprene rubber [24], etc. The anti-aging effect of PPD could be seen as a chemical anti-aging effect. The surface of GO has plenty of oxygen-containing groups [16]. By chemical grafting, PPD could be grafted onto the surface of GO, and reduce GO to graphene. As a result of the reduction process, PPD could improve the interaction between GO and polymer by reducing hydrophilic groups. In this study, GO modified with *p*-phenylenediamine (GO–PPD) was incorporated into NBR to improve the thermostability and mechanical properties of NBR.

The synthesis of GO–PPD hybrid particles, and the preparation of NBR/GO–PPD composites, are presented in the first two parts. The structure of GO–PPD hybrid particles were researched by X-ray diffraction (XRD), Raman spectroscopy, and Fourier Transform infrared spectroscopy (FTIR). The morphology of NBR/GO–PPD composites was observed by scanning electron microscope (SEM). At last, mechanical properties of NBR/GO–PPD composites were investigated through tensile testing, dynamic mechanical analysis (DMA). The Flynn–Wall–Ozawa (FWO) method was used to study thermo-oxidative aging kinetics of NBR/GO–PPD composites.

## 2. Materials and Methods

### 2.1. Materials

Graphite oxide was supplied by The Sixth Element Materials Technology Co, Ltd. (Changzhou, China). The NBR used was nitrile rubber 2907E with acrylonitrile content 29%, supplied by CNPC Lanzhou Petrochemical Company (Lanzhou, China). Sulfur, zinc oxide, stearic acid, dioctyl phthalate (DOP), and ammonium hydroxide were purchased from Chengdu Kelong Chemical Reagent co. LTD (Chengdu, China). 2,2′-Dithiobis (benzothiazole) were purchased from China Aladdin Industrial Corporation. The reagents used in this study were all analytical grade.

### 2.2. Preparation of GO–PPD

Graphite oxide was exfoliated in 1000 mL deionized water under sonication to form a 10 mg/mL GO aqueous dispersion. Then, 2 mL ammonium hydroxide and 1 g *p*-phenylenediamine were added into the GO dispersion, and stirred for 4 h at 95 °C. Product was filtrated under reduced pressure and then washed by ethyl alcohol at least six times. Finally, the product was dried in a vacuum oven at 80 °C for 48 h.

### 2.3. Preparation of NBR/GO–PPD Composites

The basic formulations of the NBR composites were described as in Table 1. The above ingredients were mixed on a two-roll mill (Guangdong Zhanjiang Rubber and Plastic Machinery Factory, Zhanjiang, China). The curing characteristics were determined by a disc oscillating rheometer (Beijing Huanfeng Mechanical Factory, Beijing, China). Then, the compounds were cured in a press vulcanizer (Shanghai Rubber Machinery Factory, Shanghai, China) under a pressure 10 MPa at 150 °C for 30 min.

### 2.4. NBR and NBR/GO–PPD Composites Aging Experiment

The NBR samples were cut into a dumbbell shape with thickness of 2 mm, width of 6 mm, and a middle standard length of 25 mm. Thermo-oxidative aging experiments were carried out in a Mingzhu 401-A aging test chamber at 90 °C for 18 days.

### 2.5. Instrumentation

Tensile tests were carried out using an MTS CMT6104 universal tester by following a Chinese national test standard of GB/T528-1998. The finial value of the test was taken as the average of at least 5 results.

FTIR spectra of GO and GO–PPD in transmission mode on compressed KBr pellets were recorded using a Thermal Scientific spectrometer (Thermo Fisher Scientific, Waltham, MA, USA). Attenuated total reflectance-Fourier transform infrared (ATR-FTIR) spectra of the NBR composites were recorded using a Thermal Fisher Scientific Nicolet iS50 attenuated total reflectance-IR spectrometer over a wavenumber range of 4000–500 cm^−1^, with 32 scanning times at a resolution of 4 cm^−1^.

XRD (PANalytical Co. X′Pert PRO, Almelo, Netherlands) was used to test the structure change of GO after modifying with PPD. During the experiment, a Cu target was selected, and Kα ray, whose wavelength was 0.154 nm, was chosen to sweep continuously at step length of 4°/min. The scanning angle was 5~70°.

Raman spectroscopy was carried out on a DXR (Thermo Fisher Scientific, Waltham, MA, USA) with a laser wavelength of 532 nm.

The morphology of the hybrid particles and NBR composites were observed using a JSM-7500F field-emission (SEM) (Japan Electronics Co., Tokyo, Japan).

The thermal analysis was carried out on a Thermogravimetric Analyzer /Differential Scanning Calorimetry (TGA-DSC) calorimeter (MettlerToledo Co., Zurich, Switzerland). Each sample weighing about 7–10 mg was heated from 50 to 800 °C at different heating rates (5 K/min, 10 K/min, 15 K/min, and 20 K/min) under an air flow of 50 mL/min.

DMA was performed on a TA Q800 apparatus (TA Instruments, New Castle, DE, USA) equipped with liquid nitrogen cooling instrument. DMA was in double cantilever beam mode from −60 to 20 °C at a heating rate of 3 °C/min and frequency of 1 Hz.

The equilibrium swelling method was used to calculate the crosslink density of the NBR composites. Samples were swollen in toluene at room temperature for 72 h and then removed from toluene and the surface toluene of samples was cleaned. The samples were quickly weighed and then dried in a vacuum at 80 °C for 36 h to remove the solvent, and then reweighed. The volume fraction of NBR in the swollen gel, $V_{0}$, was calculated by equation:

(1) $$V_{0}=\frac{m_{1}\times \varphi \times \left(1-\alpha \right)/\rho_{r}}{m_{1}\times \left(1-\alpha \right)/\rho_{r}+\left(m_{2}-m_{3}\right)/\rho_{s}}$$

where $m_{1}$ is the sample mass before swelling, $m_{2}$ and $m_{3}$ are sample masses before and after drying, respectively, φ is the mass fraction of rubber in the NBR composites, α is the mass loss of the NBR composites during swelling, and $\rho_{r}$ and $\rho_{s}$ are the nitrile rubber and solvent density, respectively.

The elastically active network chain density, $V_{r}$, which was used to represent the whole crosslink density, was then calculated by the well-known Flory–Rehner equation [25]:

(2) $$V_{r}=-\frac{ln\left(1-V_{0}\right)+V_{0}+\chi V_{0}^{2}}{V_{s}\left(V_{0}^{1/3}-V_{0}/2\right)}$$

where $V_{0}$ is the volume fraction of NBR in the swollen gel and $V_{s}$ is the solvent molar volume (107 cm^3^/mol for toluene). χ is the interaction parameter between NBR and toluene.

## 3. Results and Discussion

### 3.1. Characterization of Filler Particles

**Figure 1:**
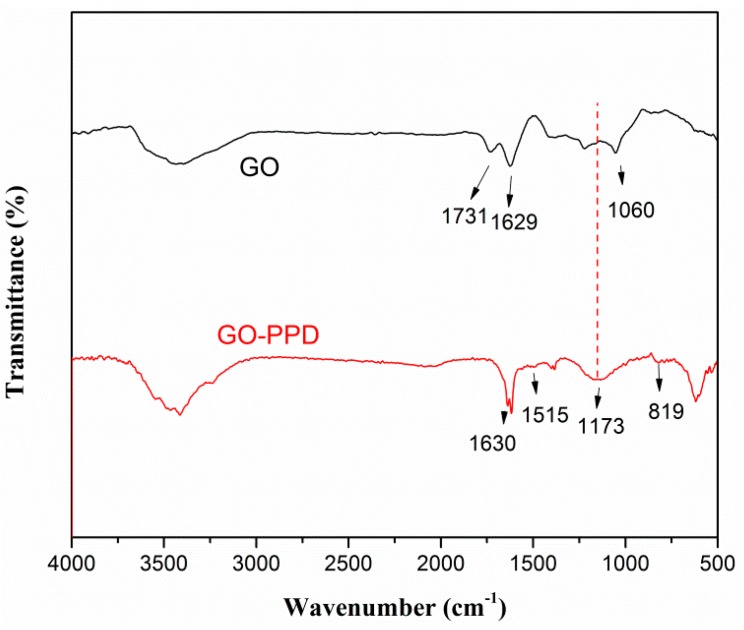
FTIR spectroscopy of GO and GO–PPD.

The reduction and functionalization of GO are confirmed by FTIR spectroscopy (Figure 1). As presented by the curve of GO, the broad peak at 3442 cm^−1^ can be attributed to the stretching vibration of hydroxyl groups (–OH). The characteristic adsorption peaks at 1731, 1629, and 1060 cm^−1^ correspond to the carbonyl stretching vibrations of –COOH groups, aromatic C=C stretching vibrations, and C–O stretching vibrations of epoxide group, respectively [26,27]. After the reaction with PPD, the characteristic absorption bands of –COOH, and epoxide groups of GO were weakened or disappeared, implying the reduction of GO by PPD. Furthermore, a new peak at 1173 cm^−1^ (C–N stretching vibration) appeared, indicating the formation of C–NH–C bands due to the chemical grafting of PPD onto GO surface via nucleophilic substitution reaction between the amine group of PPD and the epoxide group of GO [23]. This conclusion is also supported by the presence of new peaks at 819 and 1515 cm^−1^ which is attributed to the deformation and stretching of N–H in –C–NH_2_ groups, respectively [28].

**Figure 2:**
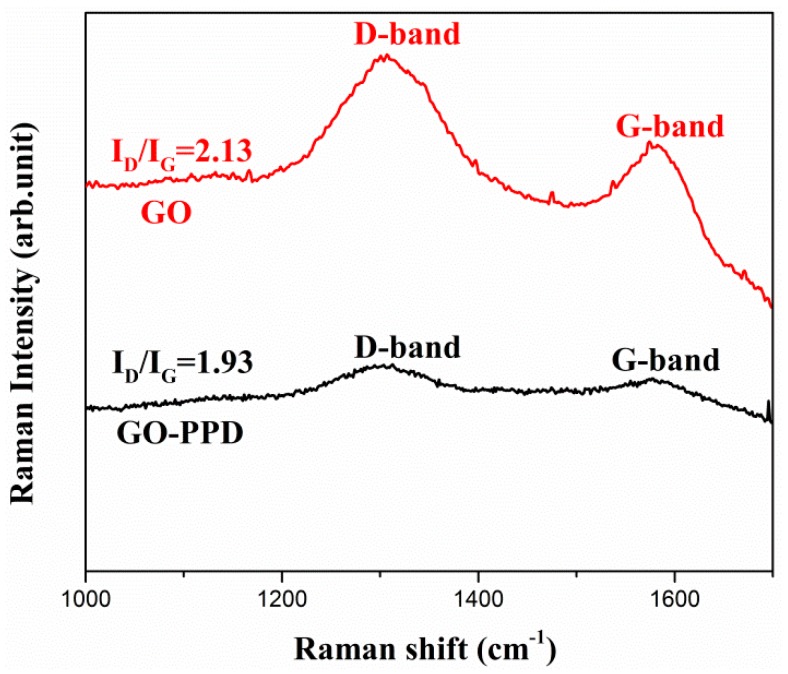
Raman spectra of GO and GO–PPD hybrid particles.

The Raman spectra of GO and GO-PPD contain two bands: D at 1312 cm^−1^ and G at 1582 cm^−1^ (as shown in Figure 2). The position and intensity of D bands and G bands provide information of the type of defects appear in the graphitic material [22,26,27]. The peak intensity ratio of D band to G band (I(D)/I(G)) indicates the amount of in-plane defects. I(D)/I(G) ratio (2.13) of GO was higher than that of GO–PPD (1.93), suggesting GO–PPD had less defects [26]. This was due to the reduction of GO after adding PPD. The amine group of PPD reacted with the oxygen-containing functional groups of GO, decreasing the in-plane defects of GO.

**Figure 3:**
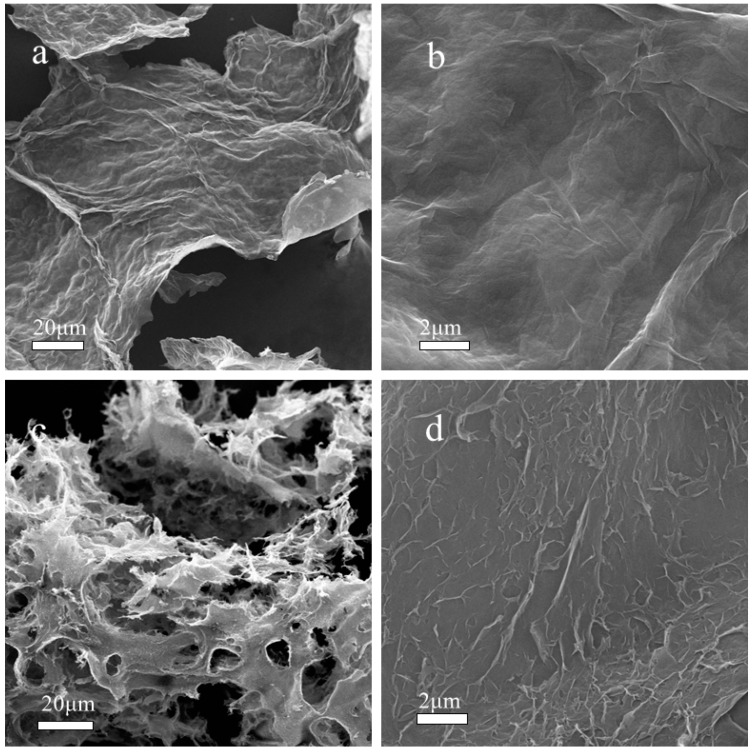
SEM photographs of (**a**,**b**) GO and (**c**,**d**) GO–PPD.

The morphologies of GO–PPD and GO were analyzed for better understanding the influence of *p*-phenylenediamine on GO in Figure 3. Both GO and GO–PPD sheets show a multilayer overlapping structure. GO showed a smooth surface and crimped margin structure, while the GO–PPD exhibited a ridged structure and displayed wrinkles on surface. The ridged structure was proposed to improve the mechanical properties of rubber. In the preparation of hybrid particle, the volume of GO–PPD was noted to be obviously larger than that of GO. As a result, GO–PPD might enhance the tensile strength of rubber more effectively than GO [29,30].

### 3.2. Tensile Properties before and after Aging

**Figure 4:**
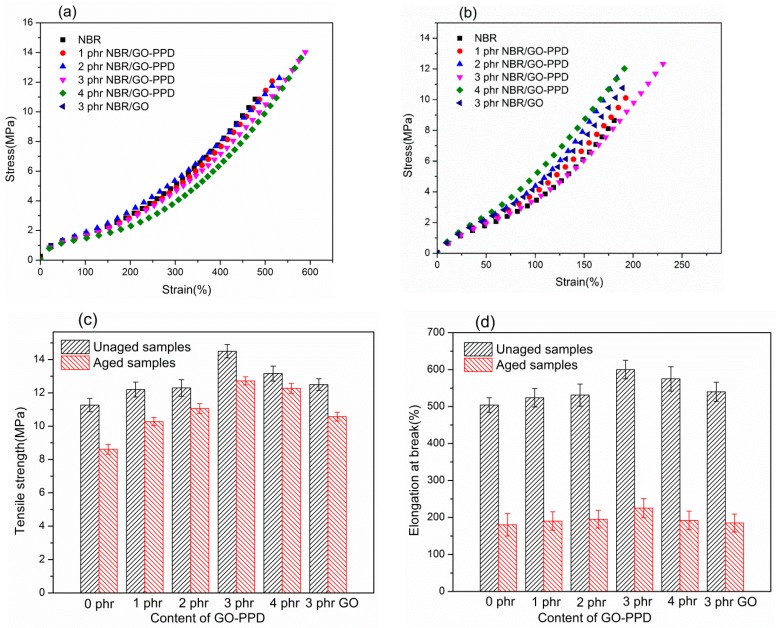
(**a**) Stress vs strain curves of unaged samples, (**b**) stress vs strain curves of aged samples for 15 days, and (**c**,**d**) tensile strength and elongation at break of different filled amounts of NBR composites before and after aging.

Tensile tests were performed on NBR composites (as shown in Figure 4). After adding GO–PPD, NBR composites showed a significant improvement in tensile strength, when the results of tensile strength were compared to the unfilled NBR. When the content of GO–PPD was 3 phr, the mechanical properties of sample were the best optimum, compared to others (as shown in Figure 4a). Even though there was 20 phr carbon black of formula for NBR (Table 1), the tensile strength and the elongation at break of NBR/GO–PPD (3 phr) composites still increase 29% and 20%, compared to NBR, respectively. In addition, compared with the mechanical properties of NBR/GO composites, the mechanical properties of NBR/GO–PPD are improved to a higher extent at the same loading of the fillers, due to the ridged structure of GO-PPD.

Furthermore, after the accelerative thermo-oxidative aging for 15 days, the retention ratios of tensile strength of aged NBR/GO–PPD composites are higher than the aged NBR. When the content of GO–PPD is 4 phr, a 95.3% in retention ratio of tensile strength is achieved (Figure 4c and Table 2). Similarly, Dong et al. have reported that the retention ratio of tensile strength of NBR is about 80 % after aging for 15 days at 80 °C [31]. Meanwhile, the elongation at break of rubber composites was also improved. Both before and after aging, the elongation at break of NBR/GO–PPD composites is better than that of NBR (Figure 4d and Table 3). Although the retention ratio of tensile strength of NBR/GO is higher than that of NBR, the retention ratio of tensile strength of NBR/GO is lower than that of NBR/GO–PPD. This indicates that GO–PPD has both physical anti-aging effect and chemical anti-aging effect in the aging process, while GO only has physical anti-aging effect.

A primary conclusion made, herein, is that the GO–PPD could not only play the role of reinforcing NBR, but also significantly improve the stability of rubber composites in the process of thermo-oxidative aging.

**Figure 5:**
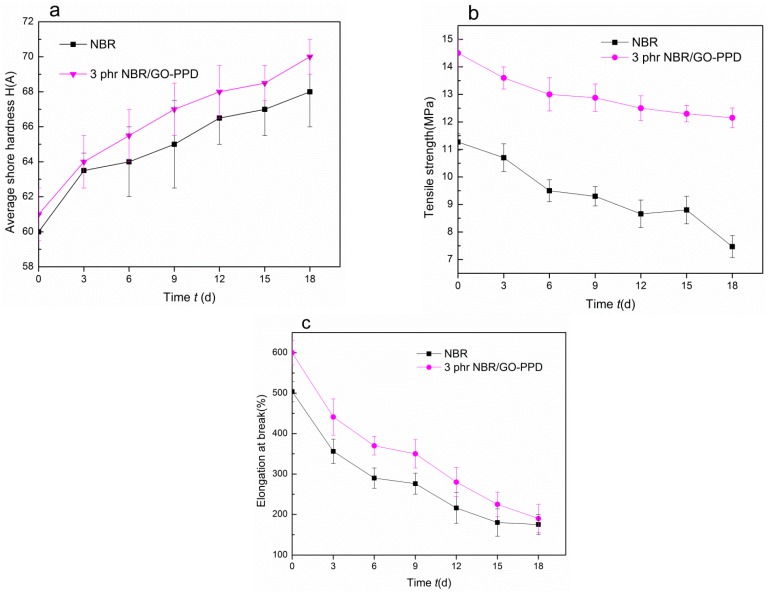
Trends in tensile property of NBR, (**a**) the average shore hardness, (**b**) the tensile strength, and (**c**) the elongation at break aged for 18 days (measured at 100 °C), subject to different contents of fillers.

According to the results of tensile tests of NBR composites, the sample that contained 3 phr GO–PPD was chosen to be compared with NBR. The changes of the shore hardness of NBR in different aging times were displayed in Figure 5a. After aging for 18 days, the shore hardness of NBR composite and NBR were both increased, and the shore hardness of NBR/GO–PPD composite was always higher than that of NBR. The decrease of tensile strength and elongation at break of NBR/GO–PPD composites were lower than that of NBR in the process of aging (as shown in Figure 5b,c). During the aging process of NBR/GO–PPD composite, the anti-aging free radical capture agent could prevent oxygen free radical from attacking the main chain of rubber [24,25].

### 3.3. Crosslink Densities of NBR Composites

**Figure 6:**
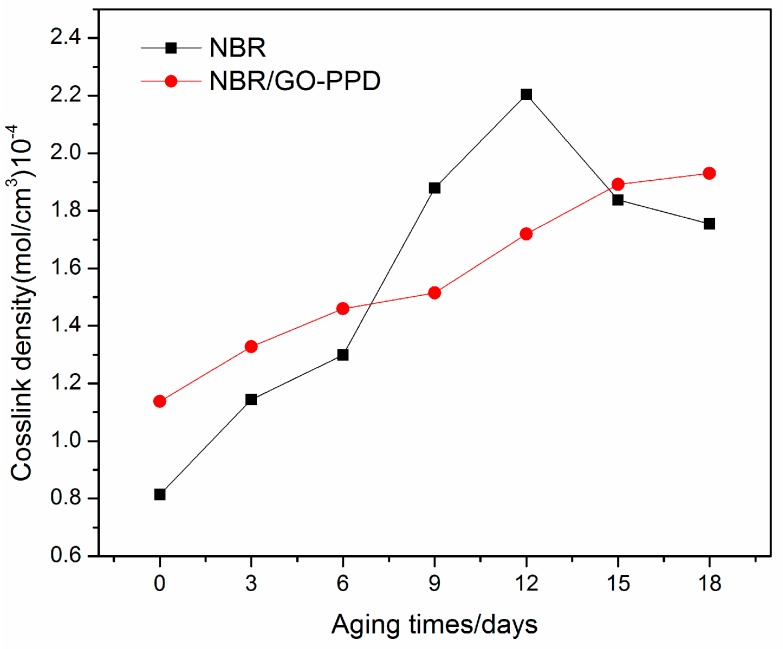
Crosslink densities of NBR composites with varying times.

The thermo-oxidative aging of nitrile rubber is believed to occur in two ways: chain crosslink and chain scission. The variation of crosslink density could reflect the process of the thermo-oxidative aging of NBR. The crosslink densities of NBR composites were shown in Figure 6. In the primary period of aging, the crosslink densities of NBR/GO–PPD were higher than that of NBR. For NBR samples, with the aging times increasing, the crosslink density quickly increased to a value about 2.21 × 10^−4^ mol/cm^3^, and then decreased rapidly. This behavior showed that in the first period of aging, the chain crosslink is dominating, and then a period of destruction of the network is followed because the chain scission played a major role in the thermo-oxidative aging process [32]. The crosslink density of NBR/GO–PPD was increased more slowly. It indicates that GO–PPD could effectively protect the network of rubber from attack by oxide free radicals.

### 3.4. Microstructure Analyzes of before and after Aged NBR Composites

**Figure 7:**
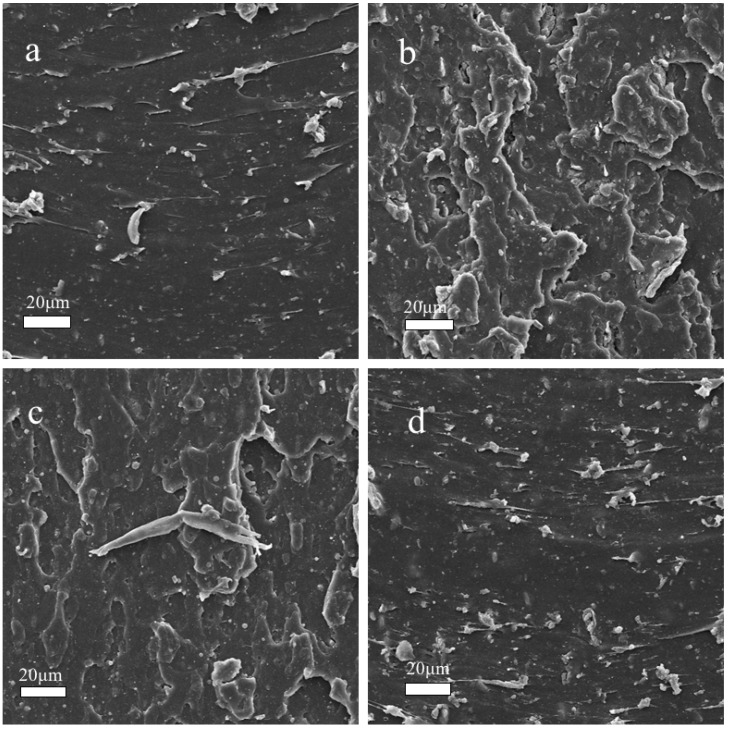
SEM photographs of fracture surfaces of NBR (**a**) before and (**c**) after aging and NBR/GO–PPD (3 phr) (**b**) before and (**d**) after aging.

The changes in mechanical properties before and after aging were caused by the crosslink and degradation of the rubber molecular chain. Variations in the surface morphology of before and after aged rubber samples were illustrated by SEM. The surface morphology of the NBR before aging was smoother than that of NBR after aging (as shown in Figure 7a,c). The surface of the neat rubber is cracked, due to oxidative attack and crosslinks of rubber. Interestingly, the surface morphology of NBR/GO–PPD before aging was cruder than that of NBR/GO–PPD after aging (Figure 7b,d), due mainly to the exfoliation behavior of GO–PPD in the rubber.

**Figure 8:**
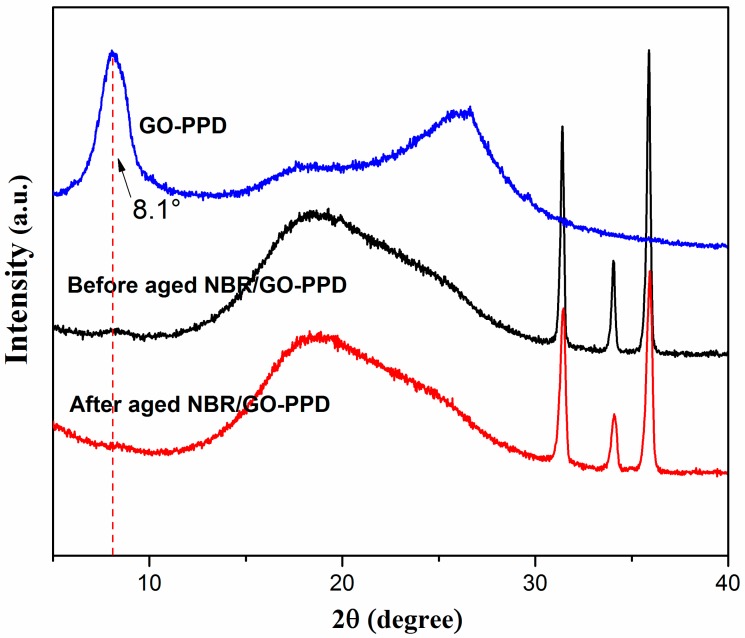
XRD diagram of GO-PPD, before aged NBR/GO–PPD and after aged NBR/GO–PPD.

XRD was used to study the exfoliation behavior of GO-PPD in the rubber (as shown in Figure 8). For before aged NBR/GO–PPD sample, the diffraction peak at 8.1°, which belongs to GO–PPD, was also present. However, for after aged NBR/GO–PPD sample, the diffraction peak of GO–PPD at 8.1° disappeared. This can be explained by that the GO–PPD exfoliation phenomenon appeared in the aging processing of rubber [4].

### 3.5. The Dynamic Mechanical of Different Contents of Composites

**Figure 9:**
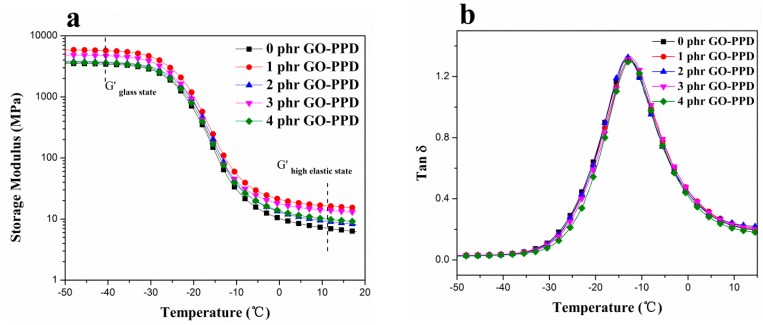
(**a**) Temperature vs G′ curves and (**b**) temperature vs tan δ curves of NBR/GO–PPD composites.

The storage modulus (G’) and loss tangent (tan δ) curves of the samples with different filler contents were shown in Figure 9, and the values details of G′ and the area of tan δ peak were listed in Table 4. With the increase of contents of GO–PPD, the value of G′ of NBR/GO–PPD composites were close to the value of 10 MPa when temperature was 10 °C and the G’ of NBR/GO–PPD composites exceeded that of NBR. In addition, the area of tan δ peak showed that the area of NBR/GO–PPD was decreased compared to that of NBR (Figure 9b). Based on the above result, we could make a conclusion that GO–PPD hybrid particles have a positive interaction with the rubber matrix [33,34,35,36,37,38,39,40].

### 3.6. Degradation Kinetics

**Figure 10:**
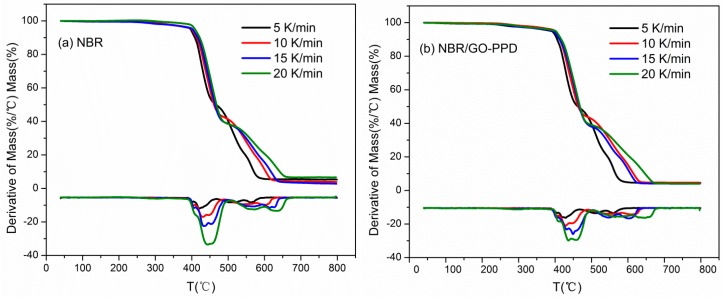
TG–DTG curves in different heating rate of two NBR composites (**a**) NBR and (**b**) NBR/GO–PPD.

The TG and DTG curves of NBR and 3 phr NBR/GO–PPD composites at different heating rates were shown in Figure 10. Although there are diverse heating rates, the curves of two composites are similar, which implies similar thermo-oxidative degradation mechanism. The DTG curves exhibit two peaks, so the thermo-oxidative degradation process can be divided into two stages. The first stage is at about 380–490 °C, corresponding to the thermo-oxidative degradation of NBR chains. And the second stage is further decomposition of thermo-oxidative products [41]. In addition, the peaks of DTG shift toward a higher temperature with the increase of heating rate.

The TG/DTG curves were proposed to determine the kinetic parameters of thermo-oxidative degradation by the non-isothermal Flynn–Wall–Ozawa (FWO) method [42] which is based on the Doyle approximation for heterogeneous chemical reactions, as shown in Equation (3).

(3) $$log\beta =log(\frac{A\Delta Ea}{R\cdot g\left(\alpha \right)})-2.315-0.4567\frac{\Delta Ea}{RT}$$

where *A* is a pre-exponential factor, ΔEa is the activation energy, *R* is the gas constant, α is the fractional mass loss, and g(α) is the conversional function. The reason for choosing FWO is that the reaction rate at a given fraction mass loss (α) is only a function of the temperature. As a result, for the constant fractional mass loss (α) at different heating rate, *Ea* could be evaluated through the slope of the straight line by plotting logβ vs. *T*^−1^.

**Figure 11:**
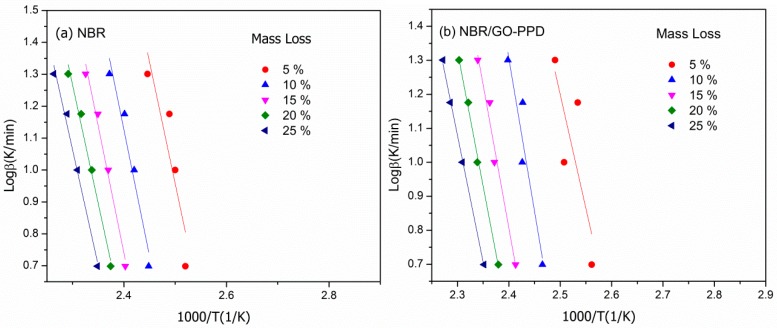
Flynn–Wall–Ozawa (FWO) analysis at specified fractional mass losses (*α*) of two NBR composites: (**a**) NBR; (**b**) NBR/GO–PPD.

The FWO fitted lines corresponding to the fraction mass losses (α) was shown in Figure 11. The relative experimental data basically fit the model, with linear correlation coefficients in the range 0.81–0.98. By the FWO equation, the slopes of fitted lines giving *Ea* at different degrees of degradation.

**Figure 12:**
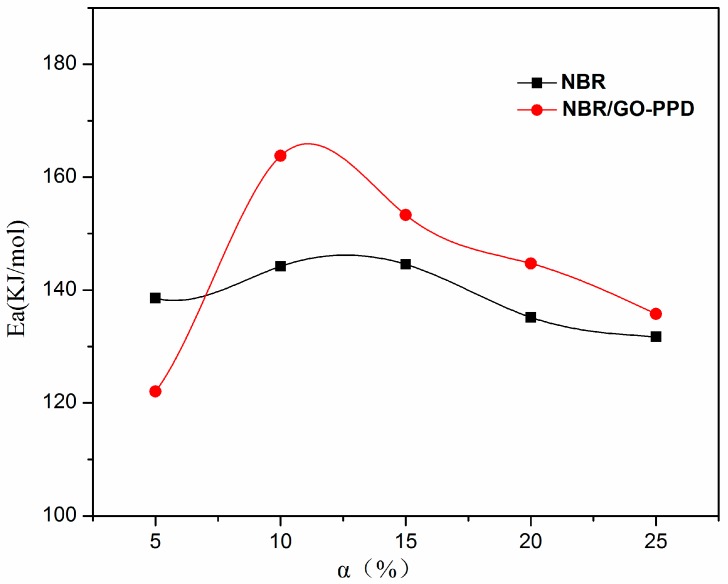
Plots of *Ea* versus fractional mass loss (*α*) determined by FWO analysis.

Figure 12 shows plots of *Ea* versus the fraction mass loss according to the FWO equation (Equation (1)). The curves could be divided into two stages. The first stage of curve, *Ea* increases with α until α reaches 10%, corresponding to the initiation of the chain [43]. For NBR, the *Ea* keeps a constant about 130–145 KJ/mol. With GO–PPD, the *Ea* is firstly at 120 KJ/mol, which is lower than the *Ea* of NBR at mass loss of 5%. After the mass loss exceeded 10%, the second stage is associated with the oxidation and degradation process of NBR composites [32]. The activation energies required for thermo-degradation of NBR/GO–PPD composites were higher than that of NBR (as shown in Table 5). It indicated that the thermo-oxidative stability of NBR/GO–PPD was superior to that of NBR.

## 4. Conclusions

This paper has demonstrated that graphene oxide has a ridged and corrugated structure after surface modification by *p*-phenylenediamine. GO–PPD has positive interactions with NBR, so that the mechanical properties of NBR are further improved. GO–PPD appeared exfoliated after rubber composite aging, due to the interaction between nanoparticles with rubber chains. Tensile strength and elongation at break of NBR/GO–PPD composites were increased compared to that of NBR. Even after aging for 15 days, the tensile strength of NBR/GO–PPD still superior to that of NBR. Especially, the retention rate of tensile strength of former could reach at least 90% when compared to 78% of the latter. The degradation kinetics study showed that GO–PPD was helpful to increase the thermo-oxidative degradation temperature and activation energies of NBR. This research gives more guidance to improving the integrated properties (mechanical properties and thermo-oxidative stability) of nitrile rubber by introducing a functional filler.

## Figures and Tables

**Table 1:** Compounding formulation of the nitrile rubber (NBR) composites.

Material	phr/NBR	phr/NBR/GO-PPD	phr/NBR/GO
NBR	100	100	100
graphene oxide–*p*-phenylenediamine (GO–PPD)	0	1/2/3/4	0
GO	0	0	3
zinc oxide	5	5	5
stearic acid	1	1	1
carbon black 234	20	20	20
sulfur	1.5	1.5	1.5
accelerator 2,2′-dithiobis (benzothiazole)	1.5	1.5	1.5
dioctyl phthalate (DOP)	1.5	1.5	1.5

**Table 2:** Tensile strength of NBR composites before and after aging.

Content of Filler	GO-PPD	GO-PPD	GO-PPD	GO-PPD	GO-PPD	GO
	0 phr	1 phr	2 phr	3 phr	4 phr	3 phr
Before aging	11.27	12.2	12.3	14.5	13.16	12.5
After aging for 15 days	8.8	10.5	11.3	13	12.54	10.8
Retention ratio	78.1%	86.1%	91.9%	89.7%	95.3%	86.4%

**Table 3:** Elongation at break of NBR composites before and after aging.

Content of Filler	GO-PPD	GO-PPD	GO-PPD	GO-PPD	GO-PPD	GO
	0 phr	1 phr	2 phr	3 phr	4 phr	3 phr
Before aging	504	524	531	600	575	540
After aging for 15 days	180	190	191	225	192	186

**Table 4:** G′(MPa) of 10 °C and the area of tan δ peak of NBR/GO–PPD composites.

The Contents of Filler	0 phr	1 phr	2 phr	3 phr	4 phr
G′(MPa) of 10 °C	7.18	16.54	9.3	14.02	10.11
Area (tan δ)	15.4	13.5	14.6	13.1	14.3

**Table 5:** The *Ea* value of NBR composites corresponding to the mass loss of thermo-oxidative degradation.

The Mass Loss (%)	5	10	15	20	25
The *Ea* value of NBR (KJ/mol)	138	145	143	135	131
The *Ea* value of NBR/GO–PPD (KJ/mol)	120	163	153	144	136
